# Reduced brain microstructural asymmetry in patients with childhood leukemia treated with chemotherapy compared with healthy controls

**DOI:** 10.1371/journal.pone.0216554

**Published:** 2019-05-09

**Authors:** Junyu Guo, Yuanyuan Han, Yimei Li, Wilburn E. Reddick

**Affiliations:** 1 Department of Radiology, UT Southwestern Medical Center, Dallas, TX, United States of America; 2 Department of Biostatistics, St. Jude Children’s Research Hospital, Memphis, TN, United States of America; 3 Department of Diagnostic Imaging, St. Jude Children’s Research Hospital, Memphis, TN, United States of America; University of Pécs Medical School, HUNGARY

## Abstract

Microstructural asymmetry of the brain can provide more direct causal explanations of functional lateralization than can macrostructural asymmetry. We performed a cross-sectional diffusion imaging study of 314 patients treated for childhood acute lymphoblastic leukemia (ALL) at a single institution and 92 healthy controls. An asymmetry index based on diffusion metrics was computed to quantify brain microstructural asymmetry. The effects of age and the asymmetry metrics of the two cohorts were examined with *t*-tests and linear models. We discovered two new types of microstructural asymmetry. Myelin-related asymmetry in controls was prominent in the back brain (89% right), whereas axon-related asymmetry occurred in the front brain (67% left) and back brain (88% right). These asymmetries indicate that white matter is more mature and more myelinated in the left back brain, potentially explaining the leftward lateralization of language and visual functions. The asymmetries increase throughout childhood and adolescence (*P* = 0.04) but were significantly less in patients treated for ALL (*P*<0.01), especially in younger patients. Our results indicate that atypical brain development may appear long before patients treated with chemotherapy become symptomatic.

## Introduction

Acute lymphoblastic leukemia (ALL) is the most common leukemia in children. Chemotherapy with methotrexate (MTX) can cure approximately 90% of patients but causes transient neurotoxicity and asymptomatic leukoencephalopathy [1,2]. Children treated with MTX are at risk for altering white matter integrity and impairing cognitive function, including processing speed, attention, working memory, and executive function [1,3–5]. Non-invasive measures of brain asymmetry may provide an indirect indicator of white matter change during chemotherapy related to neurocognitive late effects.

The human brain is approximately bilaterally symmetrical, which provides stability and the flexibility to perform different kinds of functions. However, subtle left-right structural nuances (i.e., structural asymmetries) play important roles in functional lateralization in the brain [6–9]. Some examples of functional lateralization, such as the production of speech in the left brain [10], right-ear auditory advantage [11], and right visual field advantage [10,12], have been widely studied. These lateralized functions require physical substrates. Therefore, studying structural asymmetries is critical to understanding how the human brain functions.

Structural asymmetries may be macrostructural or microstructural. Macrostructural asymmetries are macroscopic differences in shape, width, area, and volume [6,13–15] (see Fig A1 in S1 File for an example). Even though there are some correlations between macrostructural asymmetries and functional lateralization, direct causal relations between macrostructural asymmetries and lateralized functions have only rarely been demonstrated [6]. To find further direct causal relations, it is necessary to investigate microstructural asymmetry at the cellular level.

Microstructural asymmetry may bridge the gap between macrostructural asymmetry and functional lateralization in the brain. For instance, the speech areas (including Broca’s area) on the left side of the brain have more dendritic branching than do those on the right side [16], which may correspond to better signal transmission in the left-side speech areas. In addition, the patch distances of the cluster of neurons on the left in Brodmann area 22 are larger than their counterparts on the right, suggesting that there are more functionally distinct columnar systems in the left-side area 22 [17]. An electron microscopy study found thicker myelination in the left temporal lobe than in the right temporal lobe, indicating that signals are transmitted faster in the white matter of the left temporal lobe [18]. However, these studies were postmortem investigations involving a limited number of subjects, and it was difficult to differentiate the original in vivo asymmetry from that caused by cell death.

Diffusion tensor imaging (DTI) can provide indirect measures of microstructural changes and be used to examine such in vivo changes in the brain [19]. DTI measures the restricted diffusion of water in tissues and enables the orientation of fibers to be estimated; thus, DTI is sensitive to subtle changes in axons and myelin sheaths due to brain development [20], training [21], or neuropathology [22,23], and the technique has been used to detect such changes [23]. Accordingly, DTI could be an excellent tool for studying microstructural asymmetry. The technique has already been used to detect brain asymmetry with voxel-wise approaches [24]; however, because the signal is noisy, the measured asymmetry may be affected by the structural asymmetry and vary with the use of different scanners [25]. Inconsistent results for asymmetry in the arcuate fasciculus have been reported, which may indicate reliability issues with the voxel-wise approach [24,26]. A number of streamlines obtained using the DTI connectivity approach show leftward asymmetry [26]; however, this parameter lacks validation and has not been linked to the properties of white matter. Inconsistent asymmetries were reported for the auditory network in the structural connectome [27,28].

In this study, we developed a robust DTI processing procedure to overcome the above issues and increase the reliability of the results. We explored whether DTI-related metrics could detect the microstructural asymmetry in healthy child volunteers. We further investigated if and how brain microstructural asymmetries changed in patients with ALL treated with chemotherapy.

## Materials and methods

### Subjects

#### Patients with ALL

A convenience sample of 314 patients with acute lymphoblastic leukemia (ALL) sequentially treated on a uniform protocol at a single institution and completed MR imaging between 2008 and 2016 were included in the analyses (Table 1). They comprised 190 male and 124 female patients aged 1 to 19 years. None of these subjects had avid T2-hyperintense regions in their white matter. 71 patients from a total of 385 patients who were treated on the same protocol but had avid T2 hyperintensity in their white matter were excluded from the study (see Fig A2 in S1 File for an example). Patients were assigned to a low-, standard-, or high-risk group based on comprehensive risk classification, which included the presentation of clinical features, blast cell immunophenotype and genotype, and early treatment responses [2]. Patients with ALL were treated on a chemotherapy-only protocol (Total Therapy Study XVI [NCT00549848] at St. Jude Children’s Research Hospital: https://clinicaltrials.gov/ct2/show/NCT00549848). Treatment on this protocol consisted of three main phases: remission induction (6 weeks), consolidation (8 weeks), and continuation (120 weeks). MR imaging was performed post consolidation at week 7 of continuation therapy. Triple intrathecal chemotherapy with methotrexate (MTX), hydrocortisone, and cytarabine was administered immediately after diagnosis, and the dosage was age dependent. A total of four courses of high-dose MTX were administered intravenously, one every other week, during consolidation therapy. The patients with ALL under age 5 were sedated if necessary. The imaging protocols were approved by the institutional review board of St. Jude Children’s Research Hospital according to federal regulations and IRB policy. Patients or their parent or guardian were consented before starting therapy. Written informed consent was obtained from patients above 18 years of age and from parent and guardian for patients under 18. An age appropriate assent process is also followed for patients under 18 years of age.

**Table 1:** Demographic information of two cohorts.

	ALL cohort (N = 314)	ALL Age-matched subset (N = 168)	Control cohort (N = 92)	Control Age-matched subset (N = 76)
Male (%)	190 (60.5%)	99 (58.9%)	54 (58.7%)	48 (63.2%)
Female (%)	124 (39.5%)	69 (41.1%)	38 (41.3%)	28 (36.8%)
Median Age (Min, Max) (years)	6.9 (1.2,19.1)	10.6 (6.2, 19.1)	13.3 (6.2, 25.8)	11.4 (6.2, 19.1)
Low Risk (%)	150 (47.8%)	54 (32.14%)	Healthy	
Standard Risk (%)	148 (47.1%)	101 (60.12%)		
High Risk (%)	16 (5.1%)	13 (7.74%)		
Duration	2008–2016		2007–2011	

#### Control subjects

A total of 92 healthy volunteers previously recruited and imaged as controls for a separate study were included in the study (Table 1). This control population comprised 54 male and 38 female subjects aged 6 to 25 years who had not been treated with psychostimulants or psychotropic medications and were free from major physical, neurologic, or psychiatric conditions. Control volunteers were examined by MRI without anesthesia. The imaging protocols were approved by the institutional review board of St. Jude Children’s Research Hospital, and written informed consent was obtained from each subject (above 18 years) or from their parent or guardian (under 18 years) as appropriate. An age appropriate assent process is also followed for patients under 18 years of age.

### Data acquisition

MR imaging in this cross-sectional study was performed using Siemens 3T scanners (Siemens Medical Systems, Erlangen, Germany). Patient data was acquired using 20-channel head coils after high-dose MTX treatment (6 weeks in the continuation phase). Conventional imaging consisted of whole-brain axial T1w (T1-weighted) and T2w (T2-weighted) sequences with 4-mm slice thickness and an in-plane resolution of 0.82×0.82 mm. T1w images were acquired using a gradient-echo sequence, and T2w images were acquired using a turbo spin-echo sequence.

MR DTI was incorporated into the clinical protocol and performed using a spin-echo echo-planar imaging pulse sequence with 12 directions. The DTI protocol parameters were as follows: TR = 6500 ms; TE = 120 ms; b = 0, 700 s/mm^2^; 3-mm slice thickness; 40 axial slices; in-plane spatial resolution of 1.5×1.5×3.0 mm; phase encoding direction along anterior to posterior (AP) direction. DTI acquisitions were repeated four times to increase the signal-to-noise ratio (SNR). The acquisition protocol designed in 2007 was specified to acquire sufficient spatial and angular resolution data in a clinically acceptable time of approximately less than 8 minutes.

### Data analysis

Although the diffusion tensor model provides results with high precision that correlate well with axons and myelin in most cases [19,22,23], previous studies have shown that this model may be invalid in certain instances. DTI metrics may exhibit decreased reliability and specificity in cases with noisy data, signal contamination from cerebrospinal fluid (CSF) and gray matter, regions of fiber crossings, or white-matter lesions [24,26]. In this study, we developed a DTI processing procedure to ensure the reliability of our analysis by including denoising of the data, using large ROIs within a well-defined white-matter mask, and excluding patients with avid T2 hyperintensity in their white matter. Using large ROIs can increase the SNR of averaged DTI metrics and reduce the effect of fiber crossings. A well-defined mask reduced signal contamination from CSF and gray matter. There was no observed left-right bias due to gradient nonlinearity on our MR scanner.

The DTI data were first denoised by using the local principal component analysis filter [29]. The multiple acquisitions of diffusion data were manually checked for motion artifacts and corrupted acquisitions were removed by a dedicated staff. Eddy current correction was performed for each dataset using FSL. The remaining acquisitions were aligned to remove patient motion before being averaged together (FSL, https://fsl.fmrib.ox.ac.uk/fsl/fslwiki/). The processed DTI data were fitted using a nonlinear fitting algorithm in Camino software (Camino, http://camino.cs.ucl.ac.uk/). Eigenvalues were used to generate four frequently used parameter maps, including FA (fractional anisotropy), MD (mean diffusivity), AD (axial diffusivity), and RD (radial diffusivity) [23]. The two most frequently used DTI parameters, mean diffusivity (MD) and fractional anisotropy (FA), have limited sensitivity or lack sufficient specificity to differentiate subcomponents of white-matter microstructure [23]. In contrast, the other two DTI parameters, axial diffusivity (AD) and radial diffusivity (RD), can differentiate axonal and myelin injuries, with a change in AD indicating potential axonal injury and an increase in RD indicating potential myelin damage [22,23]. AD and RD are almost completely independent, but they have certain correlations with FA and MD (Fig A3 in S1 File). In healthy young subjects, the smaller AD values indicate the presence of more mature axons, whereas the smaller RD values indicate thicker myelination [20]. Therefore, AD asymmetry is referred to as axon-related asymmetry and RD asymmetry is referred to as myelin-related asymmetry.

**Fig 1:**
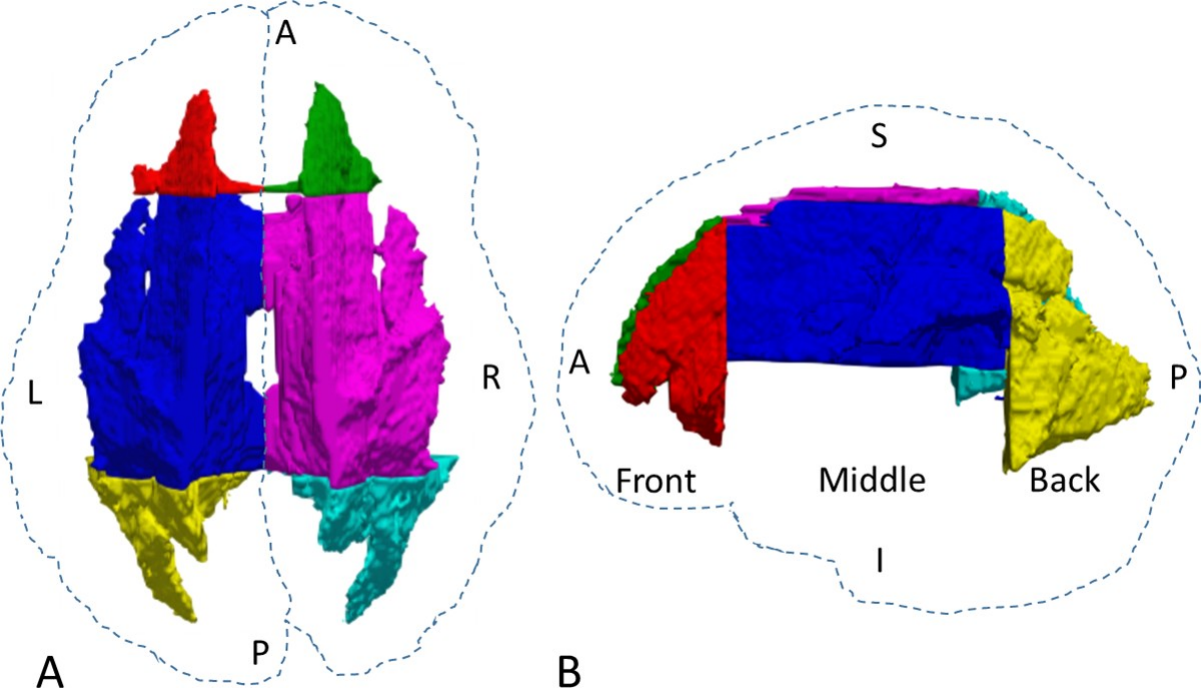
Three regions of interests in the white matter. (**A**) Top view. (**B**) Side view. The dashed contours are based on the whole brain. L, left; R, right; A, anterior; P, posterior; S, superior; I, inferior.

A white-matter mask in the standard MNI152 asymmetric atlas space was created based on the 90% white matter atlas from Johns Hopkins University [30]. The white-matter mask near the ventricle was further eroded manually to prevent issues due to slight misregistration. The white matter on the left and right sides was simply parcellated into three regions, front, middle, and back, by using two planes perpendicular to the transaxial plane and passing through the most anterior extreme of the genu of the corpus callosum or the most posterior extreme of the splenium of the corpus callosum [31]. The middle brain region was further truncated manually in the lower brain so that it contained no white matter close to the subcortical structures, thereby avoiding misregistration issues, and its volume roughly matched that of the front and back regions. The final versions of the three regions are shown in Fig 1.

MRI images were first flipped into the neurological orientation. Three-dimensional T1w images were then registered to the MNI152 asymmetric atlas by using linear and nonlinear transformations. DTI B0 and parametric images were first registered to T1w images then further registered to the MNI 152 atlas by using the transformation matrix in the T1w registration step (FSL, https://fsl.fmrib.ox.ac.uk/fsl/fslwiki/). The registered images were then checked visually and corrected if necessary. The mean diffusion parameters were calculated under the masked areas in each region.

The AI (asymmetry index) parameter was calculated using the following equation:

$$AI=\frac{P_{L}-P_{R}}{C},$$ (1)

where *P_L_* is the averaged DTI parameter on the left side, *P_R_* is the averaged DTI parameter on the right side, and *C* is the normalization constant, which is equal to 1.0×10^−3^ mm^2^/s for AD, MD, and RD and to 1.0 for FA. DTI parameters as functions of age were fitted using a logarithmic function (*y* = *A*∙*log*(*x*)+*B*) or a linear function (*y* = *A*∙*x*+*B*) to show the trend as age increased.

### Statistical analysis

A series of statistical analyses were performed to examine the relations of white matter in the different regions (front, middle, and back). All the analyses which compare ALL patient and control group were done for age 6–19 years old (Table 1). All the analyses which compare left brain and right brain within one group were done for all patients with any age (Table 1). First, for each of the DTI parameters (FA, AD, RD, and MD), we used a two-sample *t*-test to test whether there were differences between the left and right brain for the voxel-wise parameters in each of three regions of each subject. If the differences in a region did not reach significance, that region was classified as symmetric for that subject. We also used the paired *t*-test to test whether each of the region-wise parameters (FA, AD, RD, and MD) was different for the left and right brains in each cohort separately.

Next, the AI was calculated for each region in each subject. The linear mixed-effect model was used to examine whether age had different effects on the AI in different regions and similar model was used to test whether the AIs of male and female subjects differed while controlling age effect. The analyses for the patients with ALL and for the healthy volunteers were performed separately. The general linear model was used to compare the AIs of the two cohorts for each region while take multiple factors into consideration, including age, gender, group, and interaction between age and group. The general linear model was also used to examine whether the difference in AI between two locations in the control subjects differed from that in patients with ALL, after controlling age effect. Finally, the linear mixed-effect model was used to compare each DTI parameter for the two cohorts for each region while considering age, group and the interaction between age and group. A two-tailed *P*-value of less than 0.05 was considered to indicate statistical significance. The Bonferroni correction was used to adjust the multiple comparison for testing the same hypothesis in the three brain regions. Data were recorded using Microsoft Excel and analyzed using SAS v9.4.

## Results

We present only the results for AD and RD here; the results for FA and MD can be found in the Supporting information (S1 File).

### Microstructural asymmetry

The white matter in each hemisphere is subdivided into three large ROIs, front, middle, and back (Fig 1), to further diminish the noise effect after denoising. Because most areas with T2 hyperintensity in the white matter were located in the front and back of the brain [32] and the middle brain is a transition zone showing only limited microstructural asymmetry, this study focused on the ROIs in the front and back brain.

**Fig 2:**
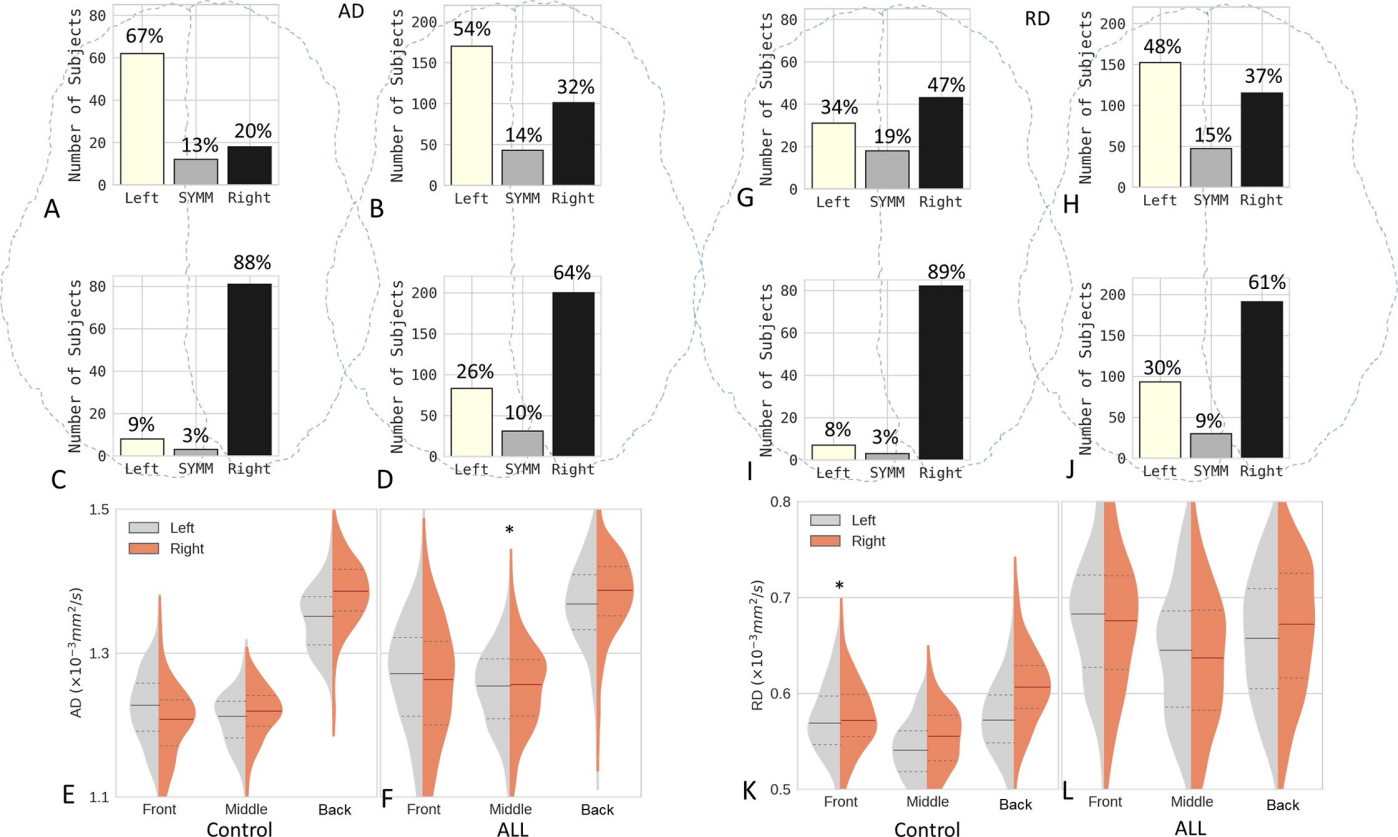
Microstructural asymmetry in the brain. (**A–F**) Axon-related asymmetry. (**G–L**) Myelin-related asymmetry. (**A, C**) The number of subjects with significantly greater axial diffusivity (AD) on one side or no significant difference (SYMM) between the two sides of the front (**A**) and back (**C**) brain in the control group (92 subjects in total). (**B, D**) The corresponding results for the ALL group (314 subjects in total). (**G–J**) The corresponding results for radial diffusivity (RD). (**E, F**) Violin plots of the average AD in each ROI for the subjects in each group, showing the kernel density estimation for each side, with bars representing the first quartile, median, and third quartile. (**K, L**) The corresponding results for RD. The medians on the left and right (**E, F, K, L**) were significantly different except for those at positions marked with an asterisk (*), which indicates no significance. ALL, acute lymphoblastic leukemia.

In the control cohort, the percentage of subjects with a larger left-side AD in the front brain (67%) was dramatically greater than the percentage with a larger right-side AD (20%) (Fig 2A). The asymmetry was reversed in the back brain, with 9% of subjects having a larger left-side AD versus 88% having a larger right-side AD (Fig 2C). As shown in Fig 2E, the mean AD value was significantly larger for the left front brain than for the right front brain but significantly larger for the right back brain than for the left back brain using two-sample t-tests, indicating that the axon-related asymmetry was flipped from front to back. Similar asymmetries were found in the ALL cohort (Fig 2B, 2D, and 2F). In contrast, there was a substantial difference in RD between the left and right back brain and a smaller difference between the left and right front brain in both cohorts (Fig 2G–2L), indicating that myelin-related asymmetry in the back brain is more distinct than that in the front brain.

### Asymmetry changes with age

**Fig 3:**
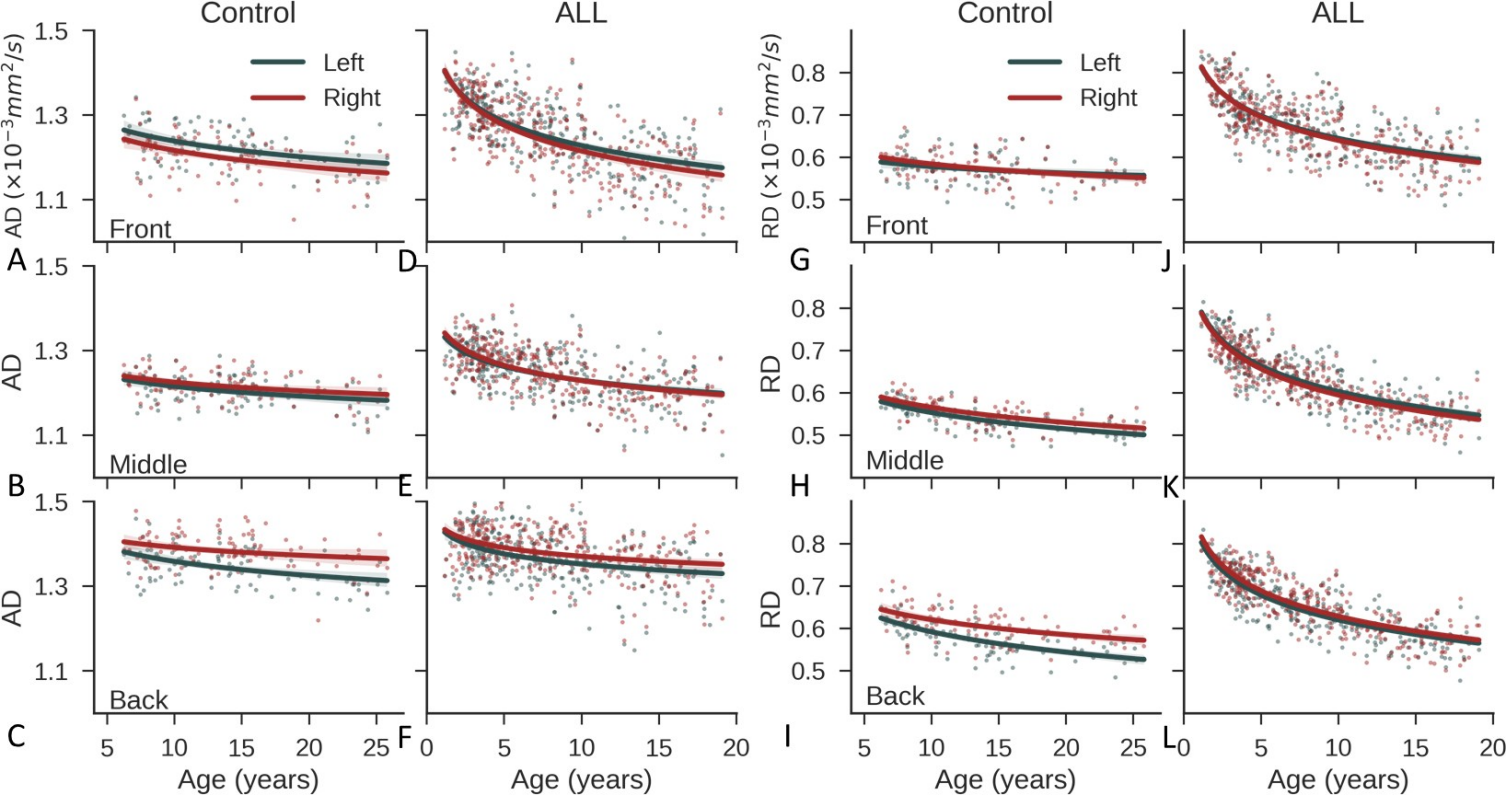
Diffusion parameters as a function of age. (**A–C**) Changes with age in the axial diffusivity (AD) on the left and right sides in the front, middle, and back brains of the control group. (**D–F**) The corresponding results for the ALL group. (**G–I**) The corresponding results for radial diffusivity (RD). The solid lines show the linear fits of the data with a logarithm function. ALL, acute lymphoblastic leukemia.

Both AD and RD decreased during brain maturation (Fig 3), which is consistent with a previous report [20]. In the control cohort, the left-right difference in AD in the front brain remained stable as age increased (Fig 3A), whereas the left-right difference in AD in the back brain increased with age (Fig 3C), and this difference was more substantial than that in the middle brain (Fig 3B). In the ALL cohort, we observed similar trends for AD, but there was less left-right difference (Fig 3D–3F). RD showed a large left-right difference only in the back brain of the controls (Fig 3G–3L).

**Fig 4:**
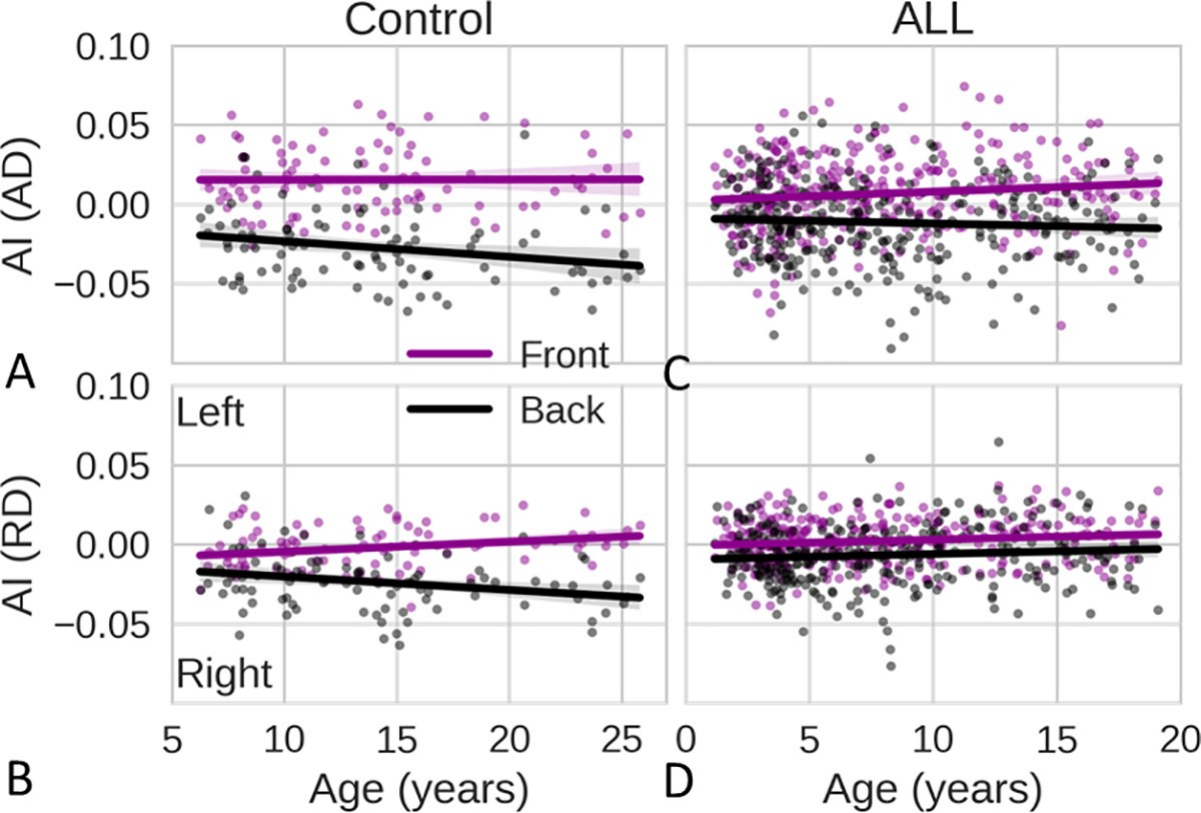
Microstructural asymmetry index (AI) as a function of age. (**A**) Changes with age in the AI of axial diffusivity (AD) in the front brains (purple) and back brains (black) of the control group. (**B**) The corresponding results for the AI of radial diffusivity (RD) in the control group. (**C, D**) The corresponding results for the ALL group. ALL, acute lymphoblastic leukemia. AI > 0 indicates leftward asymmetry; AI < 0 indicates rightward asymmetry. The solid lines show the linear regression fits of the AI data.

We compared the asymmetry indices (AIs, defined in the Online Methods) of the front and back brain (Fig 4). In the control cohort, the front brain had leftward axon-related asymmetry with a positive AI for AD, whereas the back brain had rightward axon-related asymmetry with a negative AI for AD (Fig 4A). Axon-related asymmetry in the front brain was established at an early age (<6 years) and remained stable thereafter, even though axons in the front brain continued to mature (Fig 3A–3C). Axon-related asymmetry (the AI of AD) in the back brain became more rightward with increasing age [raw *P* = 0.03, adjusted *P* = 0.09, estimate = −0.001, adjusted confidence interval (CI): (−0.003, 0.000)] (Fig 4A). The back brain had rightward myelin-related asymmetry with a negative AI for RD, and this became increasingly rightward [raw *P* = 0.01, adjusted *P* = 0.04, estimate = −0.001, adjusted CI: (−0.002, 0.000)] (Fig 4B). Myelin-related asymmetry (AI of RD) in the front brain became more leftward with increasing age [raw *P* = 0.01, adjusted *P* = 0.04, estimate = 0.001, adjusted CI: (0.000, 0.002)].

**Fig 5:**
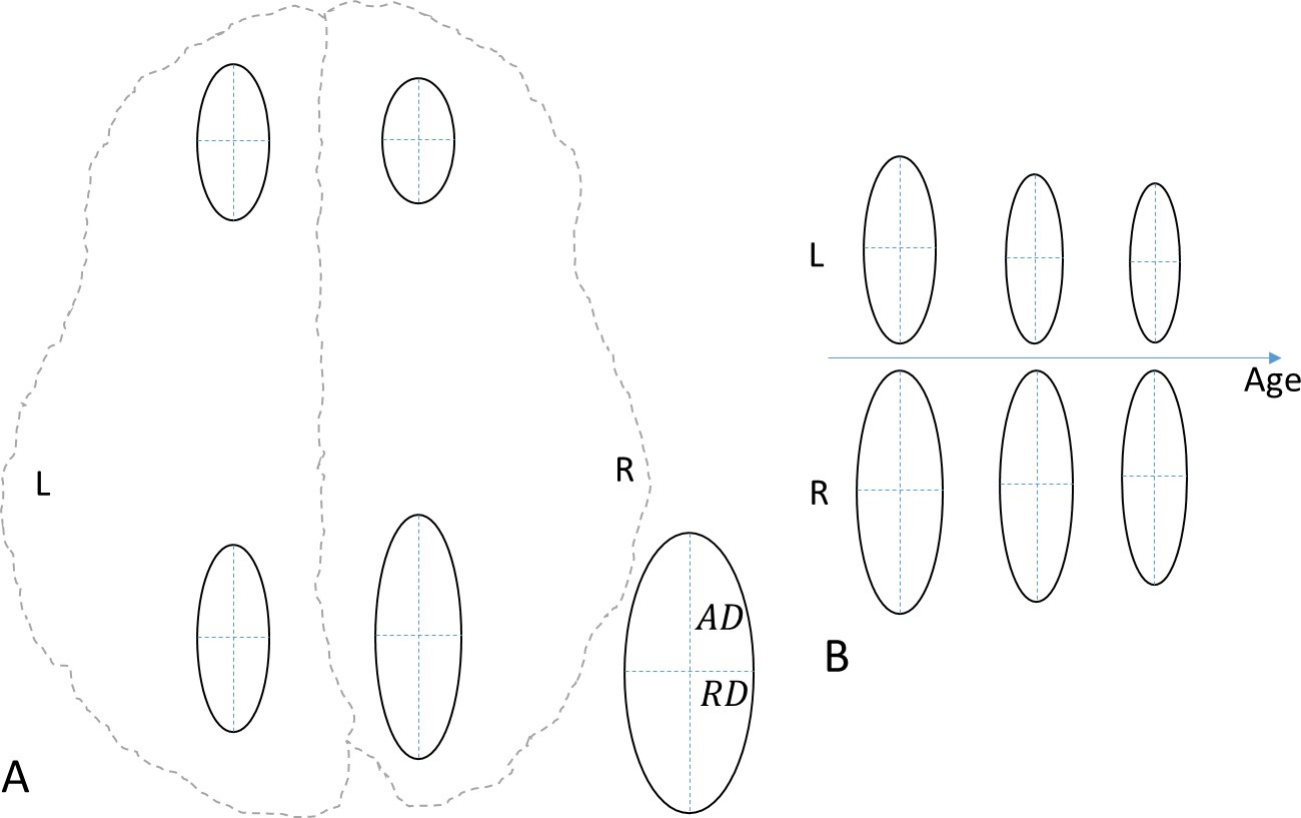
Schematic diagrams of the microstructural asymmetry of simplified diffusion tensors represented by ellipses. (**A**) The front brain shows leftward asymmetry, and the back brain shows rightward asymmetry. (**B**) Asymmetry increases with age in the back brain. AD, axial diffusivity; RD, radial diffusivity; L, left; R, right; A, anterior; P, posterior. These diagrams are based on summaries of the results shown in **Figs 2–4**.

To summarize these asymmetries, schematic diagrams of the simplified DTI ellipses with RD and AD as the radii on the x- and y-axes were drawn within a brain contour (Fig 5A), based on the relations shown in Figs 2–4. It can clearly be seen from Fig 5A that the microstructural asymmetry is flipped from leftward in the front brain to rightward in the back brain and that the ellipses in the front brain are smaller than those in the back brain, indicating that the front brain may be more mature than the back brain. Fig 5B shows how the ellipses in the back brain change with increasing age. Whereas both the AD and RD values decrease, the microstructural asymmetries continue to increase.

### Less asymmetry in patients

Chemotherapy damages the white matter of the brain [1,3]. Axon- and myelin-related asymmetry in patients with ALL treated with chemotherapy including MTX were substantially degraded. The statistical tests for the two cohorts were based on the data for participants within the same age range of 6 to 19 years. Age and interaction between age and cohort are included in the model. The following tests of the cohort effect were evaluated at the mean age of participants. The absolute mean of AI values for the AD of the ALL cohort (Fig 4C) was significantly smaller than that for the AD of the healthy cohort (Fig 4A) [raw *P* = 0.04, adjusted *P* = 0.11, estimate = −0.009, adjusted CI:(−0.018, −0.001) for the front brain; raw *P*<0.01, adjusted *P*<0.01, estimate = 0.007, adjusted CI: (0.007, 0.026) for the back brain]. The differences in the AIs of the AD between the front brain and back brain in the ALL cohort were significantly smaller than the corresponding values in the control cohort [raw *P*<0.01, adjusted *P*<0.01, estimate = −0.026, adjusted CI: (−0.038, −0.013)]. The controls had greater axon-related asymmetry in both the front and back brain when compared to subjects in the ALL cohort. The absolute mean of the AI of the RD of the back brain for the ALL cohort (Fig 4D) was significantly smaller [raw *P*<0.01, adjusted *P*<0.01, estimate = 0.024, adjusted CI: (0.016, 0.031)] than that for the control cohort (Fig 4B). The differences in the AIs of the RD between the front brain and back brain for the ALL cohort were significantly smaller than the corresponding values for the control cohort [raw *P*<0.01, adjusted *P*<0.01, estimate = −0.014, adjusted CI: (−0.023, −0.005)]. The control subjects had greater myelin asymmetry in the back brain when compared to the subjects in the ALL cohort.

**Fig 6:**
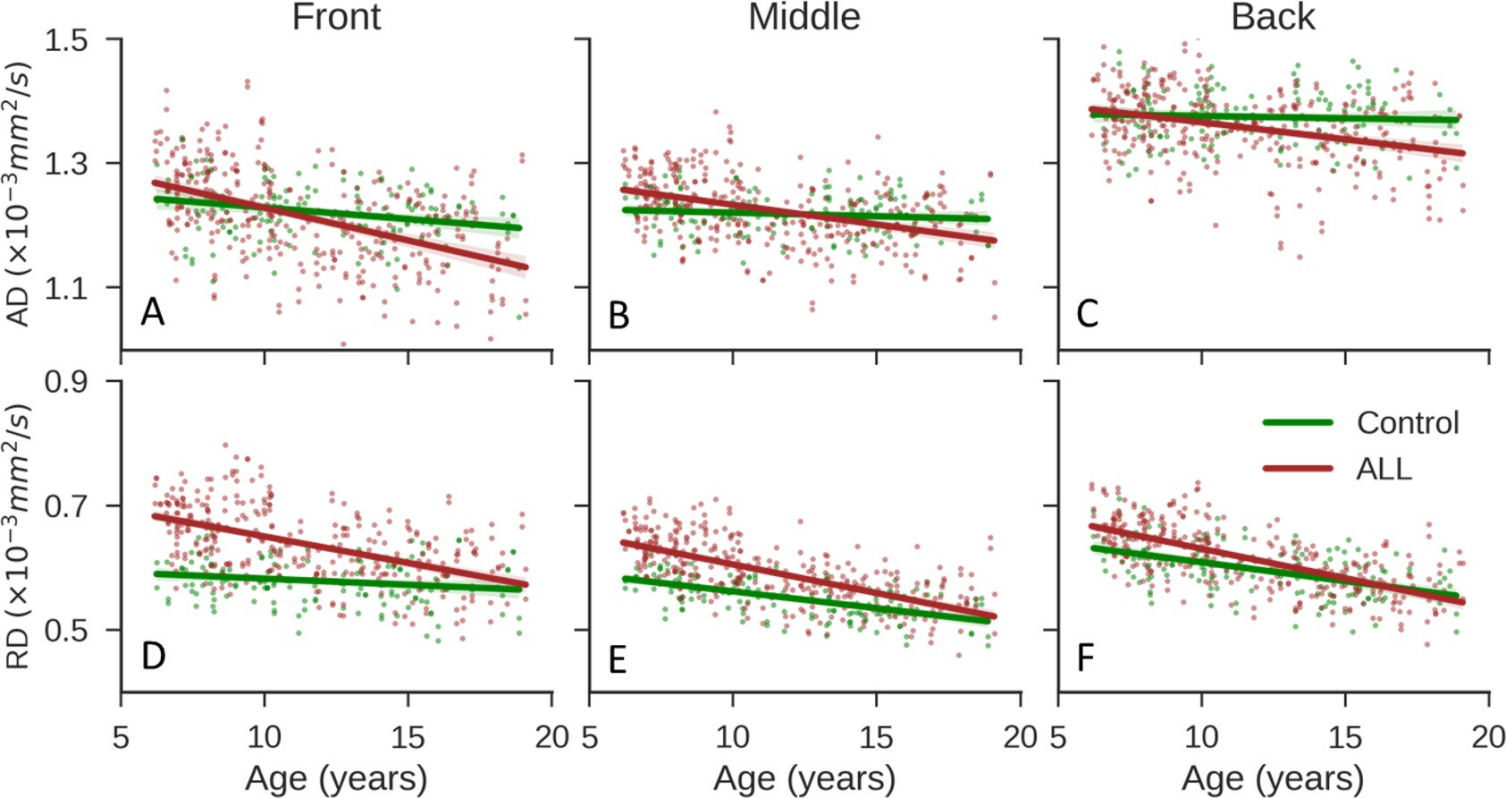
Comparisons of diffusion parameters for the control and ALL groups in three regions. (**A**) Axial diffusivity (AD) in the front brain in the control (green) and ALL (red) groups. The dots include data for the left and right sides for subjects in each group. (**B, C**) The corresponding results for the middle and back brain. (**D–F**) The corresponding results for radial diffusivity (RD). The solid lines show the linear fits of the data. ALL, acute lymphoblastic leukemia.

We plotted the AD and RD values for both sides of brain together (Fig 6) and compared the results for the controls and the patients with ALL using the linear mixed effect model. Most of the AD values were not significantly different for subjects in the two cohorts in the same age range (6–19 years of age; 169 patients and 76 controls). In contrast, the RD values of subjects in the ALL cohort were significantly larger [*P*<0.01, estimate = 0.09, CI: (0.060, 0.121)] than those of subjects in the control cohort, indicating that RD was more sensitive to brain damage and that chemotherapy might cause more damage to myelin than to axons. On comparing the two cohorts, we found that the difference in the RD in the front brain was much larger than that in the back brain (compare Fig 6D and 6F), which might indicate greater myelin damage in the front brain. The ALL cohort has faster decrease of AD and RD in front brain, middle brain and back brain when age increases compared with control group (All p values < 0.02). Finally, the RD in younger patients with ALL differed from that in controls to a greater extent than did the RD in older patients with ALL (Fig 6D). These differences decreased with increasing age, indicating that younger ALL patients treated with chemotherapy may experience more severe damage.

## Discussion

### Factors affecting asymmetry

#### Chemotherapy effect

Chemotherapy with MTX has been associated with acute injury to progenitor cells and damages white matter, which further impairs cognitive function [1,3,33]. Many clinical publications have indicated that MTX causes transient neurotoxicity and asymptomatic leukoencephalopathy in white matter [3,32]. In this study, we have shown that patients with ALL treated with chemotherapy demonstrate less microstructural asymmetry, as compared with that in healthy controls. This is the first report showing that cancer and its treatment may be associated with abnormal brain asymmetry.

In addition, survivors of ALL are at risk for neurocognitive deficits associated with their treatment. Survivors of ALL who were younger than 10 years at diagnosis had significantly lower estimated IQ (intellectual functioning) and processing speed when compared with survivors who were aged 10 years or older at diagnosis [33]. This finding indicates that younger patients may be at greater risk for deficits as a result of their treatment and is consistent with our observation that the younger patients in our study had more myelin damage than did the older patients when compared to controls (Fig 6D).

Patients with leukoencephalopathy have a larger region with avid T_2_ hyperintensity in their front brain than in their back brain [32]. This is consistent with our observation that patients with ALL, even those without avid T2-hyperintense regions, show a larger difference in myelination in their front brain than in their back brain when compared to healthy controls (Fig 6). Taken together, these findings indicate that chemotherapy with MTX may cause more damage to the front brain than to the back brain and that it may also cause neurocognitive deficits with few symptoms. These findings support the development of personalized treatment and early intervention, especially for younger patients, to reduce the incidence and severity of cognitive impairment.

#### Age effect

Differences in cortical-surface sulcal asymmetry have been reported in groups of children, adolescents, and adults [34]. There have been no previous reports of age effects on microstructural asymmetry. In this study, we have shown that microstructural asymmetries increase with age. The development of structural asymmetry promotes functional lateralization; therefore, this structural asymmetric growth is consistent with the development of functional lateralization in the brain (e.g., language development) [8]. We have studied the development of microstructural asymmetry during childhood and adolescence, but it will also be of interest to investigate the age-related changes in microstructural asymmetry in adult brains.

Factors such as sex, handedness, disease, genetics, and environmental factors [35] may also affect the microstructural asymmetry of the brain. In this study, we found no significant difference between male and female subjects. Genetic factors may contribute to the early development of handedness and language lateralization [35]. However, non-genetic factors such as age, treatment, and environment may play vital roles in structural asymmetry and functional lateralization. In this study, we have shown that microstructural asymmetry in the brain varies with age and location.

### Macrostructural asymmetry

Macrostructural asymmetries have been measured using imaging techniques such as CT and MRI [14,15]. The right frontal and left occipital petalias are two of the most prominent brain asymmetries, with the surface of one hemisphere protruding markedly beyond that of the opposite hemisphere [36]. The right frontal region has a larger volume than the left hemisphere, and the left occipital region has a larger volume than the right occipital region [6]. The frontal and occipital regions show flipped right-left asymmetries [37], which can easily be observed with the naked eye (see Fig A1 in S1 File).

Myelination is one way to facilitate signal transmission [38,39]. The myelin sheath wraps around the axon to enable much faster signal transmission with less energy consumption. Another way to expedite signal transmission is to increase the diameter of the axon [39]. However, thicker axons occupy more space in the brain, where space is limited. The combination of myelin sheath formation and axon thickening must balance the requirements for rapid signal transmission, energy efficiency, and space efficiency [39]. Because mature and myelinated axons have increased thickness, with more microtubules, neurofilaments, and vesicles to facilitate polarization, transmission, and transportation [38,40], the white-matter volume must increase during maturation, which is consistent with the finding that the total white-matter volume increases over the first two or three decades of life in humans [20].

We have shown that the front brain displays axon-related asymmetry, whereas the back brain displays axon- and myelin-related asymmetry (summarized in Fig 5). Smaller AD and RD values correspond to more mature white matter in healthy subjects [20]. White matter maturation corresponds to a white matter volume increase [41,42]. Therefore, the lower AD in the right front brain indicates that axons on the right side are more mature than those on the left, implying that there is a larger white-matter volume in the right front region. In contrast, the lower AD and RD in the left back brain indicate that the back brain on the left has more mature axons and more extensive myelination when compared to the back brain on the right, implying that there is a larger white-matter volume in the left back brain. Our findings are consistent with the macrostructural asymmetries observed in previous studies [6,14,15,37], and they provide further insights at the microstructural level.

### Functional lateralization

Each hemisphere has its own perception and specialized functions [10]. Most people have their speech-control function on the left side of their brain [10], and the right ear is better at picking up rapid speech, which is processed by the left auditory cortex, whereas the left ear is better at picking up slow musical tones, which are processed by the right auditory cortex [11]. Studies of right visual field advantage have shown faster responses in the left hemisphere than in the right hemisphere with respect to word and tool recognition [10,12,43]. The white matter in the back brain connects the auditory and visual cortices. We found that white matter in the left back brain was more mature and myelinated than that in the right back brain. Because more mature and myelinated white matter makes axonal signal transmission faster [38,39], the left back brain is more appropriate for processing faster visual and auditory signals, which is consistent with the auditory and visual functional asymmetries mentioned above [11,43].

Higher-order thinking, such as inference and creativity, is performed in the prefrontal cortex. One kind of higher-order thinking is precise and determinant and is lateralized in the left prefrontal cortex; the other kind is imprecise and abstract and is lateralized in the right prefrontal cortex [44]. Axon-related asymmetry in the front brain provides a physical substrate for this functional lateralization. Axons in the right front brain are more mature than those in the left front brain, and the right frontal volume of white matter is larger [6,14,15,37], which indicates that axons in the right front brain may be thicker and have more microtubules than those in the left front brain. This axonal structural difference may account for the different kinds of higher-order thinking on each side. In contrast, myelination may play a limited role in this functional lateralization, because the myelin-related asymmetry in the front brain is not obvious.

### Limitations

In this study, we did not investigate the effect of handedness on microstructural asymmetry because of a lack of handedness information from both cohort of patients. However, since left-handed subjects should historically represent approximately 10% of subjects, our findings are mostly based on right-handed subjects. Previous studies reported that there is no significant association of handedness with brain structural asymmetry (e.g. surface area or cortical thickness) [13,45]. If left-handed subjects had the opposite microstructural asymmetry in comparison with the right-handed subjects, the asymmetries found in our study would become even more substantial by excluding the left-handed subjects. Although the asymmetries found in this study are valid even including the left-handed subjects, whether left-handedness is reflected in different microstructural asymmetry requires further investigation. We show that microstructural asymmetries in the patients with ALL treated with chemotherapy was substantially degraded. Previous clinical studies have shown that chemotherapy causes major damage to white matter [1,3,32]. Leukemia does metastasize to the nervous system, but it rarely involves the brain parenchyma [46]. To the best of our knowledge, there are no published reports of leukoencephalopathy in a patient before chemotherapy. Although MTX is the major factor in the changes in white matter, ALL itself may also have slight effects on brain asymmetry that could not be investigated here because of the limitations of this cross-sectional study. In addition, brain asymmetry develops quickly in infancy and early childhood, but the present study could not investigate the changes during those life stages because of the minimum age of the volunteers.

### Potential applications

Brain microstructural asymmetry, as measured with DTI, may provide a biomarker for changes in brain asymmetry. The AI can be used to monitor changes due to treatment or disease. We have shown that the AI of ALL subjects receiving chemotherapy with MTX differed significantly. For asymptomatic patients without leukoencephalopathy, post-chemotherapy interventions that could lessen cognitive late effects include both pharmacologic (e.g., methylphenidate, donepezil) [47,48] and non-pharmacologic methods, such as therapist‐delivered cognitive remediation or computerized cognitive training [49–51]. Brain asymmetry may potentially be used to monitor and evaluate the results for those interventions. This method may be used to measure changes in asymmetry during other chemotherapy or radiation therapy regimens and may provide further insights into the risks associated with those therapies. Some diseases, such dyslexia, schizophrenia, and Alzheimer disease, are reportedly associated with macrostructural changes in brain asymmetry [52–54]. We believe that measuring microstructural changes in brain asymmetry may provide a better understanding of these diseases and facilitate their treatment.

In conclusion, we have found that there are microstructural axon- and myelin-related asymmetries in the front and back brain. These microstructural asymmetries become more prominent as the brain develops, but the microstructural asymmetries in the brains of patients with ALL treated with chemotherapy was substantially degraded. Together, these findings may help provide greater insights into functional lateralization in brain maturation and the neurocognitive deficits caused by chemotherapy.

## Supporting information

S1 File Eight supporting figures. (DOCX)
