# Supplementary material for: Reduced brain microstructural asymmetry in patients with childhood leukemia treated with chemotherapy compared with healthy controls
Source: PLoS One. 2019 May 9;14(5):e0216554. doi: 10.1371/journal.pone.0216554 (PMC6508708; doi:10.1371/journal.pone.0216554)
Supplement: S1 File — (DOCX) [file pone.0216554.s001.docx]

Reduced Brain microstructural asymmetry in patients with childhood leukemia treated with chemotherapy compared with healthy controls

Junyu Guo^1^*, Yuanyuan Han^2^, Yimei Li^2^ & Wilburn E. Reddick^3^

^1^Department of Radiology, UT Southwestern Medical Center, Dallas, TX, 75390. USA ^2^Department of Biostatistics, St. Jude Children’s Research Hospital, Memphis, TN 38105. USA

^3^Department of Diagnostic Imaging, St. Jude Children’s Research Hospital, Memphis, TN 38105. USA

*Correspondence should be addressed to:

E-mail: [junyu.guo@utsouthwestern.edu](mailto:junyu.guo@utsouthwestern.edu)

**Supporting information:**


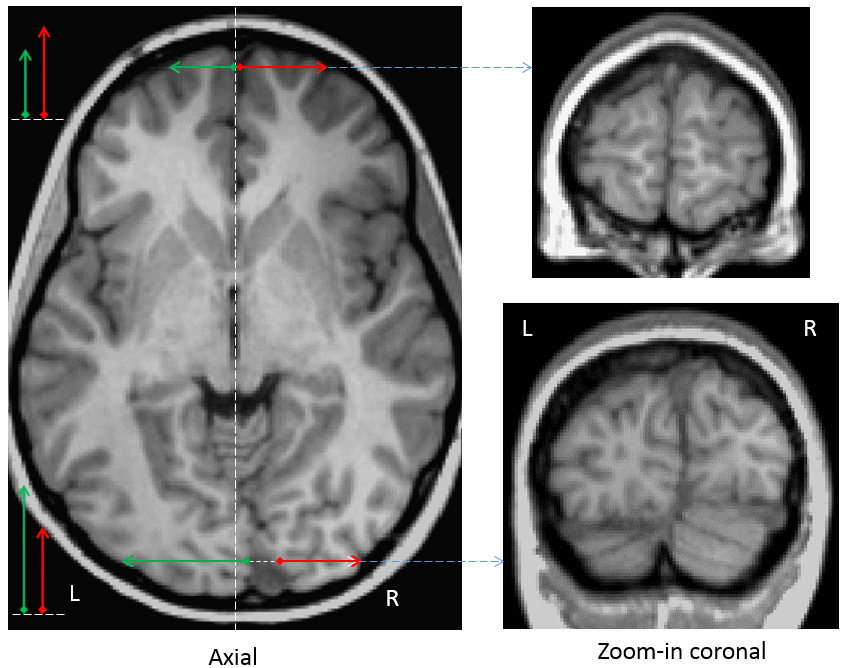


**Fig A1.**  Macrostructural asymmetry of the width of the frontal and occipital lobes in registered T1-weighted (T1w) images from a healthy female volunteer aged 14.6 years. The left image is an axial T1w image; the two right images are the coronal images at two locations marked by the two blue dashed arrows. The front brain shows rightward asymmetry, whereas the back brain shows leftward asymmetry. L, left (green); R, right (red)


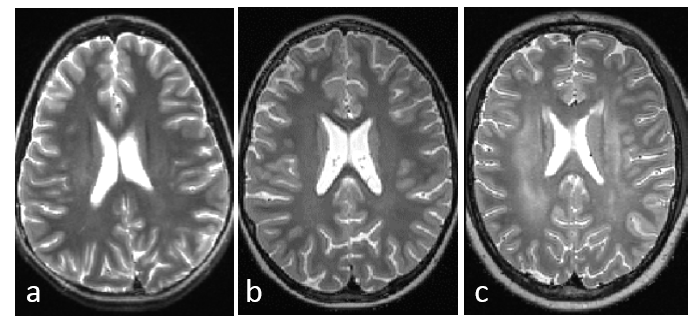


**Fig A2.** T2w images acquired at similar slice positions in three subjects. (**a**) A healthy female volunteer (aged 15 years) from the control group in this study. (**b**) A female subject (aged 12 years) with ALL but without clear leukoencephalopathy from the ALL group in this study. (c) A male subject (aged 15 years) with ALL and leukoencephalopathy who was excluded from this study. ALL, acute lymphoblastic leukemia


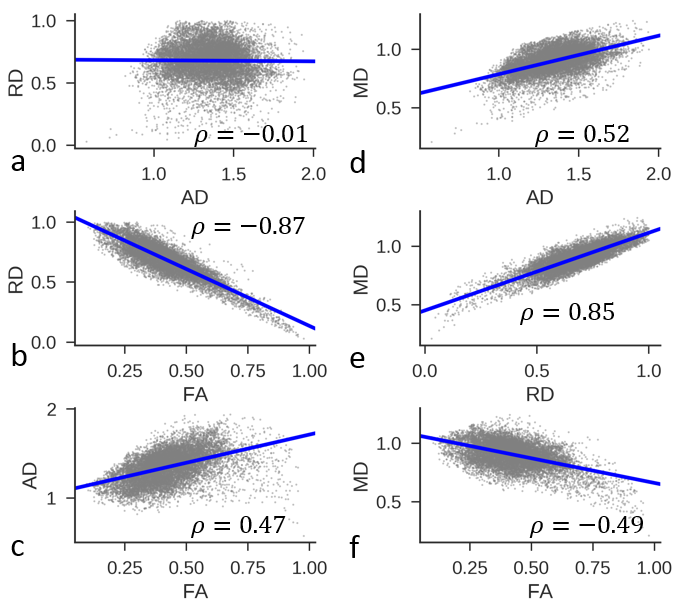


**Fig A3.**  Correlations between different diffusion parameters. (**a**) There was no correlation between radial diffusivity (RD) and axial diffusivity (AD). (**b**) There was a strong negative correlation between RD and fractional anisotropy (FA). (**c**) There was a moderate correlation between AD and FA. (**d–f**) Correlations between mean diffusivity (MD) and the other three parameters, AD, RD, and FA. The gray dots represent the data from each voxel in one region. The solid blue lines show the linear regression fits of the data. *ρ* is the Pearson correlation coefficient


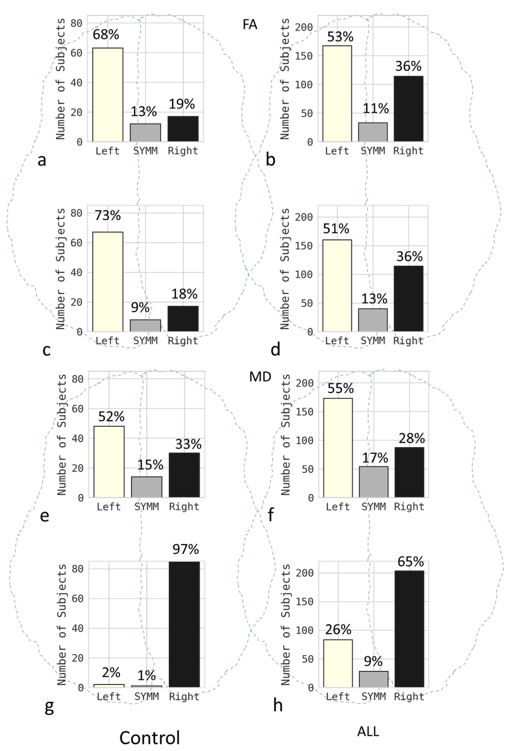


**Fig A4.**  Microstructural asymmetry. (**a, c**) The number of subjects with significantly larger fractional anisotropy (FA) on one side or no significant difference (SYMM) between the two sides of the front brain (**a**) and back brain (**c**) in the control group (92 subjects in total). (**b, d**) The corresponding results for the ALL group (314 subjects in total). (**e–h**) The corresponding results for mean diffusivity (MD). ALL, acute lymphoblastic leukemia


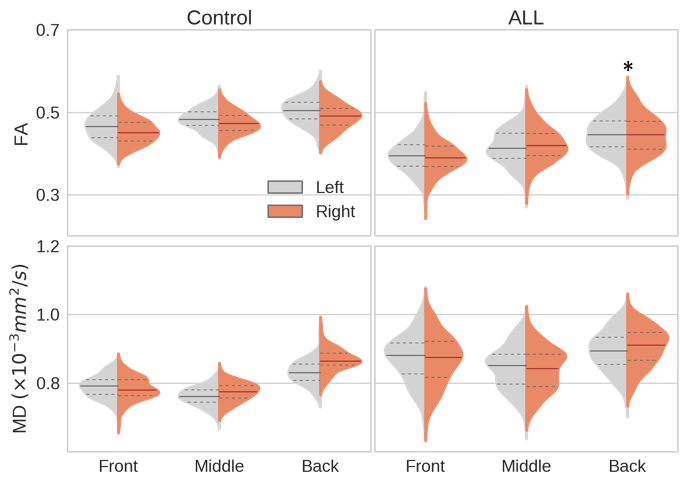


**Fig A5.** Violin plots of the average FA and MD in each ROI for the subjects in each group. The kernel density estimation is shown for each side, with bars representing the first quartile, median, and third quartile. (**a, b**) The results for the control group. (**c, d**) The results for the ALL group. The medians for the left and right sides were significantly different except for those at positions marked with an asterisk (*), which indicates no significance. FA, fractional anisotropy; MD, mean diffusivity; ALL, acute lymphoblastic leukemia


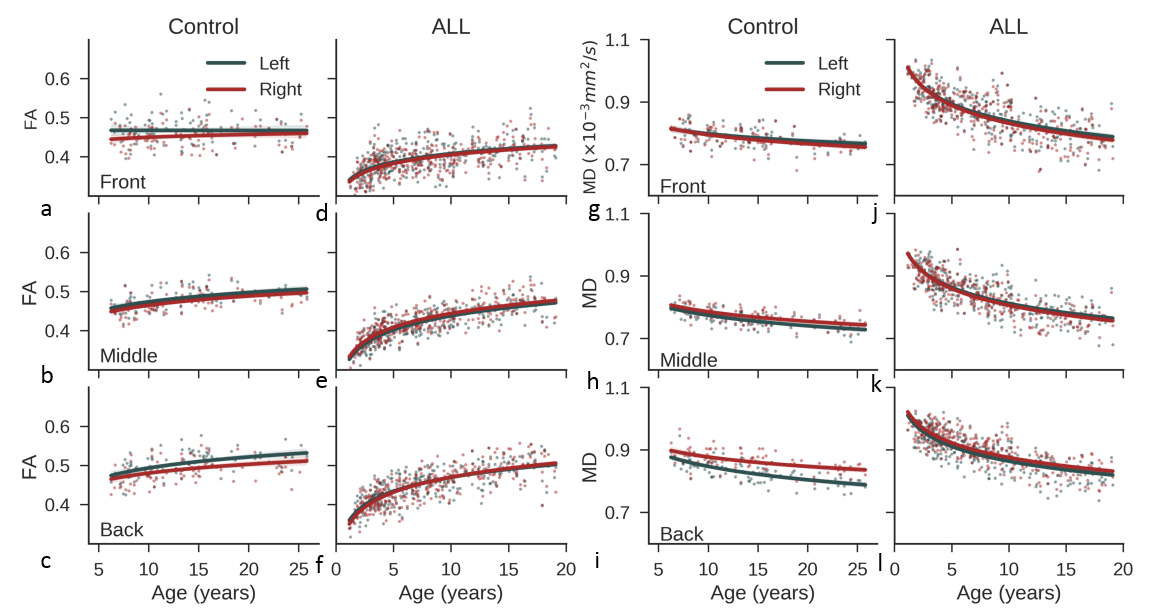


**Fig A6.**  Diffusion parameters as a function of age. (**a–c**) Changes with age in the fractional anisotropy (FA) on the left and right sides in the front, middle, and back brains of the control group. (**d–f**) The corresponding results for the ALL group. (**g–l**) The corresponding results for mean diffusivity (MD). The solid lines show the linear fits of the data with a logarithm function. ALL, acute lymphoblastic leukemia


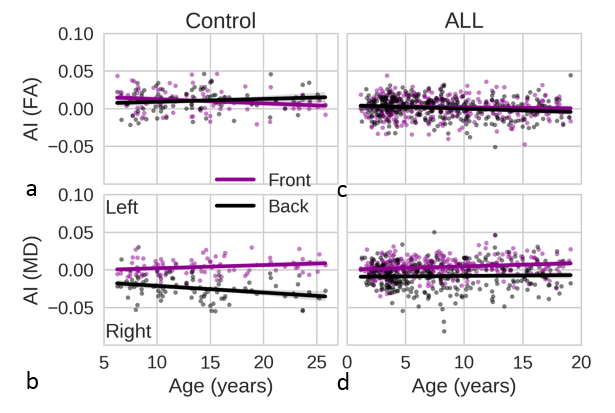


**Fig A7.**  Microstructural asymmetry index (AI) as a function of age. (**a**) Changes with age in the AI of the fractional anisotropy (FA) in the front brains (purple) and back brains (black) of the control group. (**b**) The corresponding results for the AI of mean diffusivity (MD) in the control group. (**c, d**) The corresponding results for the ALL group. ALL, acute lymphoblastic leukemia. AI > 0 indicates leftward asymmetry; AI < 0 indicates rightward asymmetry. The solid lines show the linear regression fits of the AI data


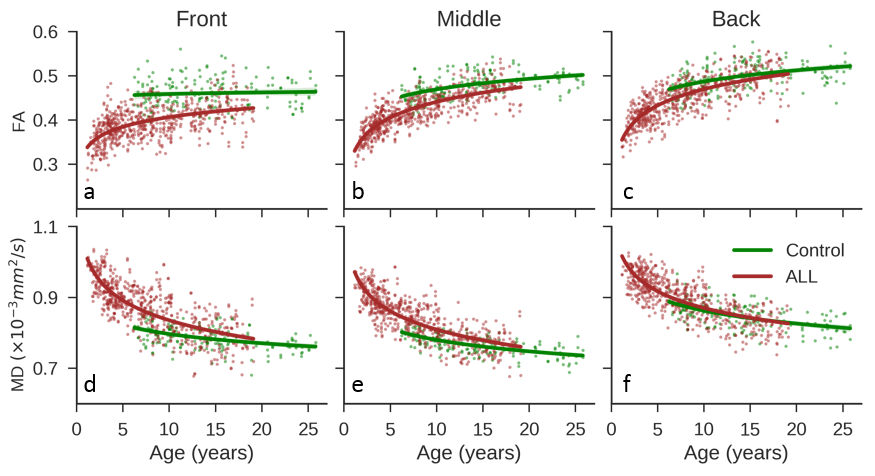


**Fig A8.**  Comparisons of diffusion parameters for the control and ALL groups in three regions. (**a**) Fractional anisotropy (FA) in the front brain of the control (green) and ALL (red) groups. The dots include the data from the left and right sides for subjects in each group. (**b, c**) The corresponding results in the middle and back brain. (**d–f)** The corresponding results for mean diffusivity (MD). The solid lines show the linear fits of the data with a logarithm function. ALL, acute lymphoblastic leukemia
